# Implant Fibrosis and the Underappreciated Role of Myofibroblasts in the Foreign Body Reaction

**DOI:** 10.3390/cells10071794

**Published:** 2021-07-15

**Authors:** Nina Noskovicova, Boris Hinz, Pardis Pakshir

**Affiliations:** Laboratory of Tissue Repair and Regeneration, Faculty of Dentistry, University of Toronto, Toronto, ON M5G 1G6, Canada; nina.noskovicova@utoronto.ca

**Keywords:** fibroblast, contracture, collagen, mechanosensing, TGF-β1, macrophage, topography, micro-pattern, elastic modulus, wound healing, tissue repair

## Abstract

Body implants and implantable medical devices have dramatically improved and prolonged the life of countless patients. However, our body repair mechanisms have evolved to isolate, reject, or destroy any object that is recognized as foreign to the organism and inevitably mounts a foreign body reaction (FBR). Depending on its severity and chronicity, the FBR can impair implant performance or create severe clinical complications that will require surgical removal and/or replacement of the faulty device. The number of review articles discussing the FBR seems to be proportional to the number of different implant materials and clinical applications and one wonders, what else is there to tell? We will here take the position of a fibrosis researcher (which, coincidentally, we are) to elaborate similarities and differences between the FBR, normal wound healing, and chronic healing conditions that result in the development of peri-implant fibrosis. After giving credit to macrophages in the inflammatory phase of the FBR, we will mainly focus on the activation of fibroblastic cells into matrix-producing and highly contractile myofibroblasts. While fibrosis has been discussed to be a consequence of the disturbed and chronic inflammatory milieu in the FBR, direct activation of myofibroblasts at the implant surface is less commonly considered. Thus, we will provide a perspective how physical properties of the implant surface control myofibroblast actions and accumulation of stiff scar tissue. Because formation of scar tissue at the surface and around implant materials is a major reason for device failure and extraction surgeries, providing implant surfaces with myofibroblast-suppressing features is a first step to enhance implant acceptance and functional lifetime. Alternative therapeutic targets are elements of the myofibroblast mechanotransduction and contractile machinery and we will end with a brief overview on such targets that are considered for the treatment of other organ fibroses.

## 1. Introduction

Implantable medical devices and body replacement parts have revolutionized modern medicine in supporting and/or replacing malfunctioning human organs and tissues. Clinical applications of implants range from tissue repair and reconstruction, prostheses, neural interfacing, biosensors, controlled drug release and electronic pacing. Despite their undisputable benefits for patients, prolonged presence of objects that are not normally part of the organism often provokes tissue repair responses that are collectively known as the foreign body reaction (FBR). The FBR often starts similar to a physiological healing response to lost tissue homeostasis following implantation surgery [1,2] and can be divided into overlapping stages, not unlike the phases that define normal wound healing: (1) protein adsorption and formation of provisional ECM, (2) acute inflammation, and (3) chronic inflammation which in the context of the FBR refers to the infiltration of leukocytes and monocytes 2–5 weeks post-implantation [3].

In this review, we will mainly discuss two subsequent FBR stages that differ from the normal wound healing process and occur at implant surfaces: (4) chronic macrophage activation and foreign body giant cell (FBGC) formation, and (5) fibroblastic cell activation into myofibroblasts that form peri-implant scar tissue—a process called implant fibrosis [4]. We will further concentrate our discussion on larger (i.e., exceeding particle sizes that do not allow cellular uptake) and ‘impenetrable’ implants that, in contrast to porous scaffold materials, do not allow cell invasion. Such solid medical devices include glucose sensors for diabetics, various drug delivery systems, artificial heart valves and pacemakers to alleviate cardiac malfunctions, orthopedic prostheses, and breast implants for plastic and reconstructive surgery [1,5,6]. For excellent overviews and commentaries on other aspects of the FBR, notably the inflammatory cell and growth factor environment, and role of vascularization, the reader is referred to these excellent recent review articles [1,2,4,6,7,8,9,10,11,12].

## 2. Clinical Complications with Implants

Implant material allergies, bacterial infections, association with cancer and tissue necrosis are some of the most frequent clinical complications associated with the FBR. For instance, exposure to metal-based materials such as those used for orthopedic, dental, or cardiac (pacemaker leads) implant devices can induce material-induced allergies [13], and cause side effects such as joint failure, pain, localized swelling, and cutaneous reactions [14]. Dental implant rejections due to poor bone quality, unresolved carries, and poor dental hygiene are most common [15]. Dental implant failures, if left untreated, can lead to other health issues, such as gum inflammation, infection, and damage to surrounding tissue and teeth [16]. In addition to implant surfaces directly causing FBR as discussed further below, mechanical overloading and implant-wear complications, for instance caused by poor implant positioning, often result in implant failures, [17]. Moreover, biological complications can be caused by the improper placing of dental implants under aseptic measures and infections caused by bacterial plaques [18].

Similarly, mechanical overloading of stainless steel-based orthopedic implants can cause clinical issues by implant loosening, ultimately leading to adverse cell responses and inflammation [19]. However, the main problems with mechanically challenged orthopedic implants such as contemporary knee and hip replacements are production of wear debris and implant corrosion accumulating over time. When joint replacements malfunction, metal and polyethylene particles can wear off the implant and trigger cell responses in the adjacent periprosthetic tissue but also spread systemically to different organs [20].

With our aging population, the demand for implantable devices is also rising to support cardiac and vascular function, such as artificial heart valves, pacemakers, catheters, stents, defibrillators, and prostheses. Cardiac implants can cause local pocket infections and ensuing FBRs that often require device extraction. Pacemakers for instance must be surgically replaced every 10–15 years to function properly and an estimated 10,000–15,000 pacemaker and defibrillators leads are extracted annually in the world [21,22,23,24]. Extraction surgery can face severe clinical complications, such as hemopericardium, major vascular injuries, and subsequent need for open cardiac surgery. As implants remain in the tissue for extended time, the formation of fibrotic tissue encapsulating the implants promotes their adhesion to major veins and cardiac structures [25]. The presence of fibrotic collagenous tissue around defective pacemaker leads increases the risk of lead extraction procedures often resulting in structural damages to vessel walls and heart tissue [21,26,27,28].

**Figure 1:**
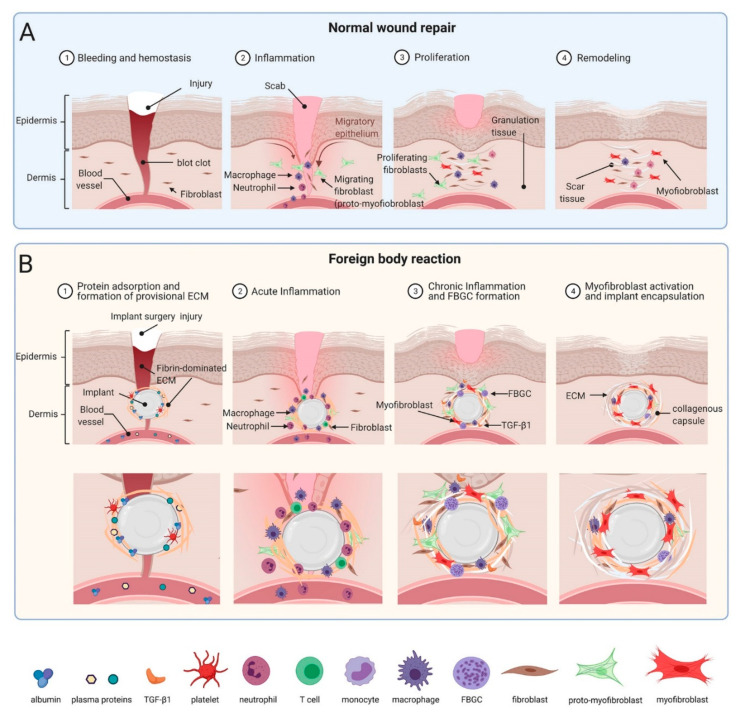
Comparison between normal wound healing stages and phases of the FBR. (**A**) Normal wound healing is compromised of the same molecular players as the foreign body reaction (FBR) to implants (**B**) In fact, the FBR begins as a normal wound healing response, but the persistent presence of the biomaterial results in sustained fibrosis and scar tissue formation. Following the initial blood-biomaterial interaction and provisional ECM formation, acute inflammation, followed by chronic inflammation and fusion of macrophages into foreign body giant cells (FBGCs) occur in a subsequent fashion. Scheme was prepared using Biorender with kind support from Ronen Schuster.

The complications associated with FBR are not limited to implantable devices used for medical or therapeutics purposes, but also in cosmetic or reconstructive surgeries. Placement of reconstructive or esthetic breast implants is one of the most common procedures performed in plastic surgery [29,30], with >330,000 implant surgeries performed in the US alone in 2020 [31]. Several complications have been reported with such procedures, including hematoma, seroma, asymmetry, scarring, swelling, and rupture [32]. However, development of a fibrotic capsule and capsular contracture remains the most common complication following breast implant surgery. While formation of an initial collagenous capsule helps positioning the implant and is usually soft and slightly firm, thicker and denser fibrotic capsules develop in >10% of the cases [33,34]. The associated capsular contraction can cause chronic pain and unwanted position changes of the implant which requires reoperation. Over 58,000 breast implant reoperations were performed in 2020 in the US due to capsular contractures [2,35]. Bacterial infections are the next most frequent complication that often require surgical revision [32]. Bacterial infection is suggested to be involved in the fibrotic reaction to implants as well as in the development of anaplastic large cell lymphoma. Breast implant-associated anaplastic large cell lymphoma is often diagnosed as a localized peri-implant seroma containing cancer cells in one breast and to the lesser degree a tumor mass attached to the surface of the fibrotic capsule. In 2011, the FDA reported that women with high-texture high-surface area implants—intended to improve tissue integration—are at the highest risk of contracting anaplastic large cell lymphoma cancer [36,37]. The main treatment option for patients is a complete surgical removal of breast implants, dissection of adjacent diseased tissue and fibrotic capsule [38]. Chemotherapy is often recommended only in patients with advanced disease stage [39]. Another complication of breast reconstruction is mastectomy-related skin flap necrosis that increases morbidity rates as it can lead to impaired wound healing, tissue scarring, infection, and ultimately implant removal and replacement [40,41]. Many of the clinical complication listed above are variations of failing normal tissue and repair processes and it seems important to briefly discuss similarities between normal wound healing and the FBR (Figure 1).

## 3. Commonalities between the FBR and Normal Wound Healing

Most if not all animal model and clinical implant placings involve surgical damage to the host tissue which inevitably creates a normal wound healing response, often starting with damage to vascularized connective tissue. Plasma proteins released form damaged vessels include albumin, complement factors, fibrinogen, fibronectin, and vitronectin. Together, these constituents form a fibrin-dominated provisional extracellular matrix (ECM), called thrombus or wound clot that stops bleeding [42]. In the presence of an implant, part of this neo-ECM adsorbs to the material surface as a 2–5 nm thick layer of provisional ECM [43,44,45] (Figure 1). In the FBR and during normal wound healing, the provisional ECM provides a structural cell-populated scaffold that stores and continuously releases various mitogens, chemo-attractants, cytokines, and growth factors, all influencing the outcome of the FBR [2,46]. In both cases, hemostasis is followed by and overlaps with a phase of acute inflammation that ranges from hours to few days and usually resolves within one week [7].

Within hours post implantation, neutrophils are among the first immune cells that appear at the tissue- implant interface and their primary task is to clear bacteria and other debris [47]. Degranulating mast cells release histamine and serotonin, causing vasodilatation which allows easier access to other inflammatory cells migrating into the wound site [48,49]. Release of factors such as interleukin (IL)-4 and IL-13 by mast cells also plays an integral role in recruiting monocytes to the site of implanted material where they differentiate into macrophages [50,51,52]. Macrophages eventually become the primary cell population around the implant and at the implant surface [53]. Even in a normally healing wound and around resolving implants, macrophages are present for multiple days to phagocytose dead cells, damaged tissue as well as possible degradation products of the implant. The failure of macrophages to digest implants whose sizes and material properties withstanding phagocytosis is one of the main causes that turn acute into chronic (not resolved) inflammation at the implant site as discussed below.

Macrophages, platelets, and other implant-adjacent cells of wounded tissue, such as keratinocytes, endothelial cells, and adipocytes secrete various profibrotic and angiogenic growth factors, including platelet-derived growth factors (PDGF), vascular endothelial growth factors (VEGF), and transforming growth factor-beta (TGF-β) which among other functions attract and activate injury-adjacent fibroblastic cells [54]. During normal wound healing, fibroblastic cells replace provisional and weak fibrin with stronger collagenous ECM to restore the lost tissue architecture and mechanical integrity. Collagen fibers produced during the normal wound healing phase of the FBR show anisotropic orientations not unlike that of healthy connective tissues such as skin dermis [55]. Overlapping with ECM production, fibroblastic cells develop contractile features that allow first migration into the provisional ECM and later organization of the neo-collagen ECM into mechanically resistant scar. The transition from a quiescent to migratory (proto-myofibroblast) to a contractile phenotype is termed myofibroblast activation [56,57] (Figure 1) and will be discussed further below in the context of implant fibrosis. Myofibroblast are main contributors to the formation of scar tissue that characterizes peri-implant fibrosis and together with their fibroblast cousins become the major cell population in the ECM around implants within 4 weeks [5]. In severe cases, the dense fibrotic capsule entirely isolates the implanted material from the local tissue environment, possibly serving as our body’s last defense mechanism against the foreign object. In contrast, remodelling and contraction of normal wound healing blends into the resolution phase, when myofibroblasts and fibroblasts undergo apoptosis, neo-vasculature decreases, and damaged tissue is repaired [58].

## 4. What Is Different between Normal Wound Healing and the FBR? The Implant

Although the onset of the FBR shares several features with normal wound healing, the nature of the implanted material has a profound impact on the progression of acute immune and repair reactions into chronic conditions [59]. Depending on their purpose, desired durability and mechanical requirements, implants come in various shapes and sizes. Our body appears to be able to repair without scarring when wounds are small (~1 cm diameter) and cell-free gaps of provisional ECM can be covered by cell migration. Implanted solid or cell-free materials increase the distance that cells must cover to populate wound granulation tissue [60]. Consequently, wounds with sizes greater than 1 cm develop scar features if treated with a biomaterial ECM, but damages up to 30 cm in depth or length can be treated without adverse fibrotic response if the biomaterial is pre-populated with cells from the wound tissue [61]. Such physical conditions may underly different FBR-susceptibilities of materials with identical molecular chemistry but different dimensionality. For instance, alginate spheres with different diameters ranging from 0.3–0.5 mm, 0.7 mm, 1 mm, 1.5 mm implanted into the peritoneal cavity of mice for 2 weeks generate stronger FBRs with decreasing sphere diameters [62]. This effect, possibly generated by the higher curvature of smaller particles, is independent from the implant material since the same results are recapitulated with spheres made of different materials, including solid glass, polycaprolactone, polystyrene, and stainless steel.

**Figure 2:**
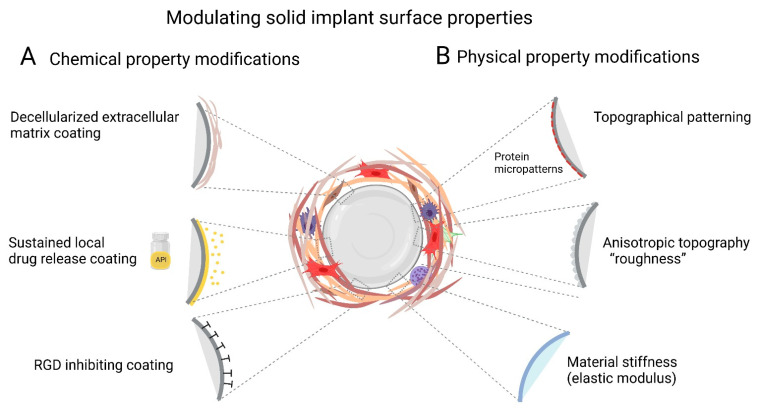
Modulating implant surface properties is a common strategy to enhance tissue integration while simultaneously reducing the incidence and severity of implant FBR and fibrosis. Protein coverage and cell interactions with the surface both determine the ability of cells to attach and spread on implants. Various physical and chemical modifications to the Implants surface are used to inhibit FBR. (**A**) Chemical modifications of surface properties include coating with local and slow releasing anti-fibrotic drugs to prevent implant fibrosis. In addition, subcutaneously implanted tissue expanders coated with decellularized ECM into non-human primates display minimal fibrotic. Inhibiting αv-integrin binding, e.g., with the RGD-peptidomimetic inhibitor CWHM-12 attenuate implant encapsulation by preventing mechanical activation of latent TGF-β1 and myofibroblasts. (**B**) In addition to or combined with altering implant surface chemistry, other treatments include modulation of physical parameters such as surface hydrophilicity or wettability, porosity, stiffness (elastic modulus), anisotropic ‘roughness’, and regular topographies. Perceived stiffness and ‘true’ modulus of the implant surface can be used to control cell responses. Restriction of focal adhesion sizes by micropatterning stiff surfaces with adhesion sized fibronectin islets limits myofibroblast spreading, intracellular stress and α-SMA stress fiber incorporation. Modulating implant geometry (size, thickness, shape, and pore size) or coating with low fouling material such as zwitterions reduces protein adsorption to implant surfaces and reduces implant fibrosis. Scheme was prepared using Biorender.

In addition to having different dimensions, implants are produced of materials with very different surface chemistries [6]. All these properties influence their biointegration potential by affecting host tissue and plasma protein adsorption—a process called Vroman effect [63]. For example, mass spectrometry analysis of proteins adsorbed at the surface of polyethylene glycol (PEG)-based hydrogels identified more than 200 different adsorbed proteins most of which are associated with wound healing and acute inflammation [64]. Protein coverage and cell interactions with the surface both determine the ability of cells to attach and spread on implants [46,64]. Therefore, modulating implant surface properties is a common strategy to enhance tissue integration while simultaneously reducing the incidence and severity of implant FBR and fibrosis. In addition to or combined with altering implant surface chemistry [65], treatments include modulation of physical parameters such as surface hydrophilicity or wettability [66], porosity [67,68], stiffness (elastic modulus) [69,70], anisotropic ‘roughness’ [71,72], and regular topographies [73,74] (Figure 2).

Surface charge has been shown to alter the efficacy of host protein adsorption to the implant. For example, the plasma proteins fibronectin and vitronectin possess a higher affinity for positively or negatively charged hydrophobic implant surfaces than to uncharged hydrophobic surfaces [75,76,77]. Moreover, it has been suggested that macrophage rather adhere to hydrophobic and cationic implant surfaces than to hydrophilic and anionic surfaces [3]. Likewise, providing implant materials with hydrophilic surfaces, for instance by oxygen plasma treatment, generally improves cell adhesion and spreading, partly by facilitating the initial absorption of ECM proteins. As a result, basic cell functions such as proliferation, survival, and differentiation are enhanced [78,79,80]. Conversely, reducing surface wettability of polyurethane, a material frequently used for medical devices, by using zwitterionic and anionic chemistry decreases macrophage spreading areas which regulates their secretion of proinflammatory cytokines and ability to fuse into FBGCs [81]. Ultra-low-fouling zwitterionic hydrogels are also to prevent peri-implant fibrosis, and promote wound healing and neovascularization in the adjacent tissue when subcutaneously implanted in a mouse model [43]. In addition to coating with zwitterions, non-fouling hydrophilic PEG is another material that reduces protein adsorption to implant surfaces [82,83]. Although one of the possible drawbacks of PEG is its susceptibility to oxidation damage, there are currently no indications of such adverse reactions in vivo [84].

Surface wettability can be changed by physico-chemical treatments and/or by modulating material porosity, which is often used to enhance the stability of implants in the target tissue [85,86]. Laser-generated micropores of ~90 µm enhance the roughness and wettability of titanium surfaces used for dental or bone implants [87], with rougher surfaces promoting spreading of osteoprogenitor cells and improving osseointegration [88,89]. Surface roughness typically refers to irregular topographies with feature sizes that can span from hundreds of millimeters down to the sub-micron level. Whether cell responses are exclusively due to the enhanced wettability and/or caused by increased surface roughness or the created topographies is unclear and combinatorial effects are likely. For instance, silicone implant materials such as those used for breast reconstructive and esthetic surgery are produced with smooth and textured surfaces, both having similar wettability properties [90,91]. Nevertheless, silicone implants with textured ‘rough’ surfaces in a micron-range show protection against implant fibrosis whereas smooth surface implants promote formation of FBGC and become encapsulated by scar tissue [3,92,93,94,95,96,97]. Similarly, matching the molecular topography of silicone implants with that of normal breast connective tissue suppresses FBR and implant fibrosis [98]. Despite their fibrosis-suppressing effect, the safety of textured breast implants has been reviewed by the FDA, as there are studies suggesting that patients with textured breast implants and surface roughness of 300 µm are at a higher risk of developing breast implant-associated anaplastic large-cells lymphoma compared to patients with smooth implants [99,100]. To mitigate such reactions, micro- and nanotextured implant surfaces have been developed with combined smooth and textured surfaces and a surface roughness of 1–100 µm [74,101]. In the range of these dimensions, surface roughness features around 4 µm were recently shown to cause minimal inflammation and FBR in different animal models and humans [74]. Another interesting approach to counteract host reactions is to provide the implant material with a stealth coat of ‘body-own’ ECM [102,103]. For example, subcutaneously implanted tissue expanders coated with decellularized ECM into non-human primates displayed minimal fibrotic encapsulation. Likewise, brain microelectrodes coated with decellularized primary astrocyte-derived ECM suppressed macrophage activation and decreased astrogliosis compared to non-coated microelectrodes [104]. However, while implant coating with human ECM is an interesting approach to mitigate the FBR, it is difficult to achieve for large implants given limited availability of autologous material, and expensive. Furthermore, although such implant surface treatments are experimentally shown to reduce fibrotic reactions but suitability in clinical applications and regulatory approvals are largely pending.

Instead of providing implant materials with a rough irregular surface, different patterning approaches have been developed to elicit defined cell responses by controlling cell attachment. Lithography methods can be applied to polymer implant materials that are shaped during the polymerization process such as polyurethanes, silicones, and various hydrogels. Numerous studies demonstrated cell responses to topographies, typically involving cell alignment and shape confinement on top of elevated structures or in the pits of the designed landscapes [73]. When arranged in highly regular geometries, topographical features can prevent force-dependent alignment of cell-ECM focal adhesions and reduce cell adhesion by preventing elongation of the spreading cells [105]. Likewise, laser-generated complex but regular surface abrasions with nanometer-deep and micrometer-wide topographical features guide the formation of focal adhesions, control cell spreading area, and direct basic cell functions of human mesenchymal stromal cells towards osseointegration of titanium materials [106]. Similar topographical patterning approaches have been used to provide innovative bulk metallic glass materials with brush-like arrays of rods with cross-sectional diameters ranging from 55 nm to 200 nm and heights of ~0.5 µm [107]. In addition to controlling cell spreading by providing different rod surface areas for focal adhesion formation, the different cross-diameters result in different rod bending stiffnesses and thus resistances to cell traction forces, not unlike the silicone micropillar substrates produced in seminal works of the Chen lab [108]. Because different cell types exhibit different pattern preferences, controlled patterning can be used to enhance implant integration by differentially regulating adhesion of distinct cell types. In the example of bulk metallic glass ‘brush’ surfaces, rods with diameters <150 nm prevent macrophage spreading and <100 nm diameter rods prevent endothelial cell spreading. Fibroblasts adhere to all arrays but exert different forces that increase with increasing rod diameters [107]. Similar effects are obtained by etching stiff titanium materials to produce pliable lamellar nanostructures that are being perceived as “soft” by seeded macrophages [109].

These and other studies indicate that perceived stiffness and ‘true’ modulus of the implant surface can be used to control cell responses. Medical implants are carefully designed and fabricated from materials that are compatible with surgical handling but can be orders of magnitude stiffer than the host tissue. Physiologically relevant elastic moduli falling into the mechanosensing range of cells are related to the stiffness of their normal tissue environments. Several studies have used atomic force microscopy to measure the Young’s elastic modulus (stress over strain, in Pa) of various organs and tissues that typically receive implants, such as skin (0.1–10 kPa) [110], brain (0.1–0.5 kPa) [111], fat tissue (1–3 kPa) [112], and liver (1–2 kPa) [113]. Muscle tissues were measured to be moderately stiffer (10–15 kPa) [114] whereas teeth and bone are considered as stiff organs (1–4 GPa) [115]. The range of stiffnesses that cells can discriminate is proportional to their contractile force and the strain they can induce in a material, which is comparably low in macrophages (≤5 kPa) and higher in fibroblastic cells (≤100 kPa) [116,117].

**Figure 3:**
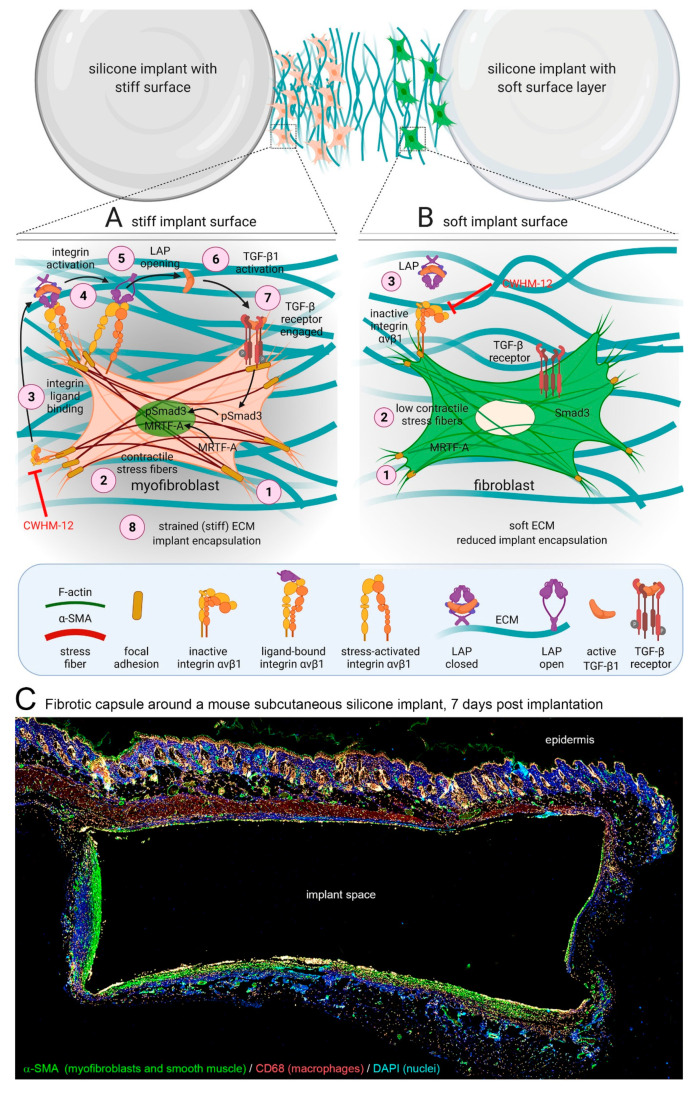
Myofibroblast mechanisms of fibrotic encapsulation of implanted stiff materials. (**A**) During the FBR, inflammatory cells secrete profibrotic cytokines which recruit fibroblasts to the implantation site. The stiff implant surface and surrounding extracellular matrix (ECM) mechanically activate fibroblasts into myofibroblasts at different levels. (1) Formation of focal adhesions with the ECM allows (2) formation of α-SMA-positive stress fibers and development of high contractile forces, evidenced by translocation of myocardin-related transcription factor (MRTF)-A from the cytosol into the nucleus. (3) Following integrin binding to ligands such as the latency associate peptide (LAP), (4) cell contraction and mechanically resisting stiff ECM enhance activation of integrins in αv integrin-containing focal adhesions, including integrin β1. (5) Mechanically activated αvβ1 integrin binds with high affinity to the LAP portion of the ECM-bound large latent TGF-β1 complex (latent TGF-β1 binding protein LTBP not shown). (6) Transmission of cell forces to the stable connection results in unfolding of LAP and release of active TGF-β1. (7) Active TGF-β1 binds to the TGF-β receptor complex, promoting phosphorylation of Smad and translocation to the nucleus. (8) Both, TGF-β1/pSmad3 and MRTF-A signaling drive pro-fibrotic programs that further enhance myofibroblast activation and encapsulation of stiff implants with a stiff ECM capsule. (**B**) Reducing implant surface stiffness and/or inhibiting αv-integrin binding with the RGD-peptidomimetic inhibitor CWHM-12 at the beginning of this cascade both attenuate implant encapsulation by preventing mechanical activation of β1 integrin, latent TGF-β1, and myofibroblasts. Scheme was prepared using Biorender. Reproduced with permission from [69]. (C) Immunostaining of a cross-section through the peri-implant tissue forming after 7 days around 2 MPa-stiff silicone disks, implanted under the dorsal skin of a mouse. Myofibroblasts (α-SMA, green) accumulate at the implant surface together with CD68-positive macrophages (red) and in deeper layers of fibrotic tissue. Diameter of the implant: 6 mm.

As a result of the mechanical mismatch between implants and host tissue, movement of the tissue results in shear stress and strain at the interface, leading to mechanical activation of inflammatory and fibroblastic cells [7,118,119]. Even breast implants that appear macroscopically soft due to their malleable content are produced with a resistant outer shell that is thousand times stiffer than the surrounding connective host tissue. Our own research has demonstrated that silicone-based soft coatings with an elastic modulus resembling that of skin dermis (~2 kPa) effectively reduce detrimental fibrotic encapsulation of stiffer (~2 MPa) silicone materials used in breast reconstruction by inhibiting activation of fibroblasts in a mouse implant model [69] (Figure 3). Considering that metal-based implants are typically used in tissues that undergo high mechanical load and wear similar to bone and teeth, coting with soft layers is not practicable in these applications. However, implants that are designed to release diffusible compounds (e.g., insulin pumps), to measure local tissue environments (e.g., glucose sensors), or to stimulate tissue (e.g., deep brain stimulation electrodes) have been provided with soft hydrogels that generally reduce FBRs and fibrotic encapsulation in rodent models [120,121,122,123,124]. In the upcoming sections, we will revisit how modulation of implant surface properties specifically affects the activity of macrophages and fibroblasts in engineering strategies to reduce FBR and implant fibrosis.

## 5. Implant Surfaces from the Perspective of a Macrophage

Macrophages are early responders to surgically placed implants and accumulate at the implantation site for multiple days to phagocytose cell and tissue debris and micron-sized implant abrasion products [125]. The presence of macrophages results in the upregulation of pro-inflammatory and pro-fibrotic cytokines such as IL-1, IL-8, monocyte chemotactic protein-1 (MCP-1), chemokine (C-X-C motif) ligand 13 (CXCL13), and macrophage inflammatory protein (MIP) [2]. These and other factors promote further macrophage recruitment and changes in macrophage activity states during the FBR [67,102]. The inability of macrophages to completely engulf large implants leads to frustrated phagocytosis and chronic inflammatory actions [7], including formation of FBGC [126], and trophic actions on vascular cells [127,128], adaptive immune cells [9,129,130], and fibroblasts [4,131]. All these actions contribute to the FBR and often to implant fibrosis. Consequently, abolishing macrophage recruitment in animal implant studies or using animals with varying degrees of immune perpetuations (i.e., genetic knockouts, chemical and/or antibody guided immune cell depletions) exhibit reduced FBR in the absence of macrophages. For instance, clodronate liposome-induced macrophage deletion blocks monocyte infiltration, FBGC formation, neovascularization, and fibrosis [125,127,132]. Moreover, targeting macrophage receptors such as colony stimulating factor (CSF)-1 receptor, which is significantly upregulated following implantation of materials, completely suppresses development of implant fibrosis [125]. In the following sections, we will focus on strategies that aim in suppressing macrophage activation by physically altering implant surface properties.

The goals of implant surface modifications targeting macrophages are at least two-fold: (1) suppressing the formation of detrimental macrophage phenotypes while stimulating regenerating and resolving activation states [133,134,135], and (2) preventing the formation of FBGCs which typically follows the chronic inflammatory phase of the FBR [126]. Pro-inflammatory macrophages are often called ‘M1’, referring to activation states that can be produced in vitro by classical activation with lipopolysaccharides (LPS) and/or interferon-γ (IFN-γ). M1-like macrophages are the most predominant polarization type present at the surface of implantable materials [67,127] and drive early FBRs, for instance by producing PDGF, tumor necrosis factor (TNF-α), IL-6, granulocyte-CSF (G-CSF), and granulocyte macrophage CSF (GM-CSF) [2,136]. In contrast, pro-regeneration (but also pro-fibrotic) ‘M2’ are generated in culture by stimulation with IL-13 and/or IL-4. It becomes amply clear that M1 and M2 are only extremes of a spectrum of different macrophage activation states and/or subtypes, including M1/M2 hybrid ‘scaffold associated macrophages’ [134,137]. IL-4 and IL-13 are also critically involved in driving macrophage fusion into FBGCs [138] suggesting that the M2-like phenotype is a precondition for the formation of multinucleated FBGCs. FBGCs are formed at implant surfaces by the fusion of macrophages in a frustrated attempt to phagocytose exceedingly large objects, accompanied by production of reactive oxygen species and ECM-degrading proteases that are detrimental to the implant and surrounding tissue structure [2,9,126,139,140].

Numerous studies investigated how macrophage recruitment, adhesion, spreading, activation, and fusion depend on implant surface chemistry and topography [11]. Macrophages primarily use integrins to attach to RGD domains in host proteins that adsorb to the surface of implants, of which αMβ2 integrin (aka Mac-1 receptor or CD11b/CD18) was one of the first receptor described in this context [141,142]. In addition, macrophages also directly attach to the body-foreign material and sense surface characteristics using Toll-like receptors (TLR) and scavenger receptors [143]. Consequently, modulating receptor engagement by modulating implant surfaces affects the ability of macrophages to attach and spread on surfaces [144] and to fuse into FBGCs [138,139,145]. In addition to integrins and RhoA/ROCK signaling [146], transient receptor potential vanilloid 4 (TRPV4) channels appear to be critical in mediating macrophage responses and polarization on differently stiff substrates [147] and macrophage fusion in a Rac1-dependent manner [148].

Spreading ability and shape directly control macrophage polarization states, i.e., their secretory and remodeling activities in addition to the expression of characteristic polarization markers [134,149]. For instance, engagement of integrins and downstream focal adhesion kinase (FAK) and Wnt signaling differentially regulate macrophage phenotypes at titanium implant materials with different degrees of surface roughness in vitro and in vivo [150]. This exemplary study adds to a large body of literature on primary and lineage macrophages and showing that providing implants with defined surface roughness and nano-/micro-topographies allows control over the transition of M1-like into M2-like macrophages by determining their spreading area and shape. The range of micro-roughness feature sizes that promote M2-like phenotypes appears to be remarkably narrow between 0.51–1.36 μm [151]. Likewise, sub-micron to single digit micron-sized surface topographies are forcing macrophage alignment, generate M2-like macrophages, and stimulate macrophage fusion into FBGCs, whereas irregular topographies with similar dimensions or larger and smaller features produce circularly spreading macrophages with M1 characteristics [152,153,154,155,156]. Another narrow window of macrophage phenotype control appears to exist at the nanometer perception level. When seeded onto bulk metallic glass implant materials with 55 nm surface patterns, M1 and M2 pre-polarized macrophages show reduced cell spreading areas, reduced pro-inflammatory profiles, higher phagocytic activities, and higher fusion rates compared to the same cells on non-patterned surfaces or surfaces with 100–200 nm features [157,158,159].

In addition to surface topographies and adhesive patterns, the stiffness of surfaces has been shown to be critical for the responses of human and mouse lineage and primary macrophages to implant materials. Stimulating cultured macrophages with LPS and IFN-γ on stiff (elastic modulus ≥47 kPa) but not on softer (≤27 kPa) polyacrylamide hydrogels elicits pro-inflammatory response in a process depending on TLR4, NF-kB, and p65 signal transduction [160]. In general, growth on ECM-functionalized soft materials results in reduced spreading areas and suppresses pro-inflammatory macrophages phenotypes compared to macrophages grown on stiffer substrates [70,161,162,163,164]. Furthermore, growth on soft culture materials reduces the ability of mouse lineage and human alveolar macrophages to phagocytose micron-sized particles [165]. Likewise, the fusion of macrophages into FBGCs is enhanced by stiffer, more adhesive and 3D versus 2D implant materials [166]. In rodent implantation models, macrophage-driven inflammation is reduced around soft versus stiffer materials or when providing the implant with a soft layer, for instance using kPa-soft PEG hydrogels [70]. Although the definition of soft and stiff can largely vary between studies, elastic moduli around and below 1–5 kPa seem to be perceived as soft by macrophages in most studies. This mechanosensation threshold is higher for fibroblastic cells which drive the formation of scar tissue around implanted materials.

## 6. A Fibroblast View on the Implant: The Origins of Implant Fibrosis

Inflammatory environment, such reactive oxygen species, phagocytosis, and/or proteolysis alone can severely impact certain implant materials that are degradable such as replacement scaffolds or depend in their function on physiological environment, such as glucose sensors. Formation of fibrotic capsules around implantable devices can additionally lead to the physical isolation of the device from the tissue which can disrupt device sensing functions, cause pain and disconnect cell-seeded implants from nutrient support [5]. Implant surface properties affect macrophage activities which in turn affects fibrogenesis in the implant environment. Materials that skew macrophages into M2-like phenotypes and/or promote fusion into FBGCs seem generally more prone to become encapsulated by fibroblast-derived scar-like ECM [4]. However, it is beginning to be appreciated that implant surface properties also directly contribute to fibrogenesis. Above, we have discussed how material adhesivity/wettability, stiffness, and topography generally affect the engagement of fibroblasts with implant surfaces surface. Next, we will focus on how implant surface properties affect implant fibrosis by exerting control over activation of fibroblastic cells into myofibroblasts.

**Figure 4:**
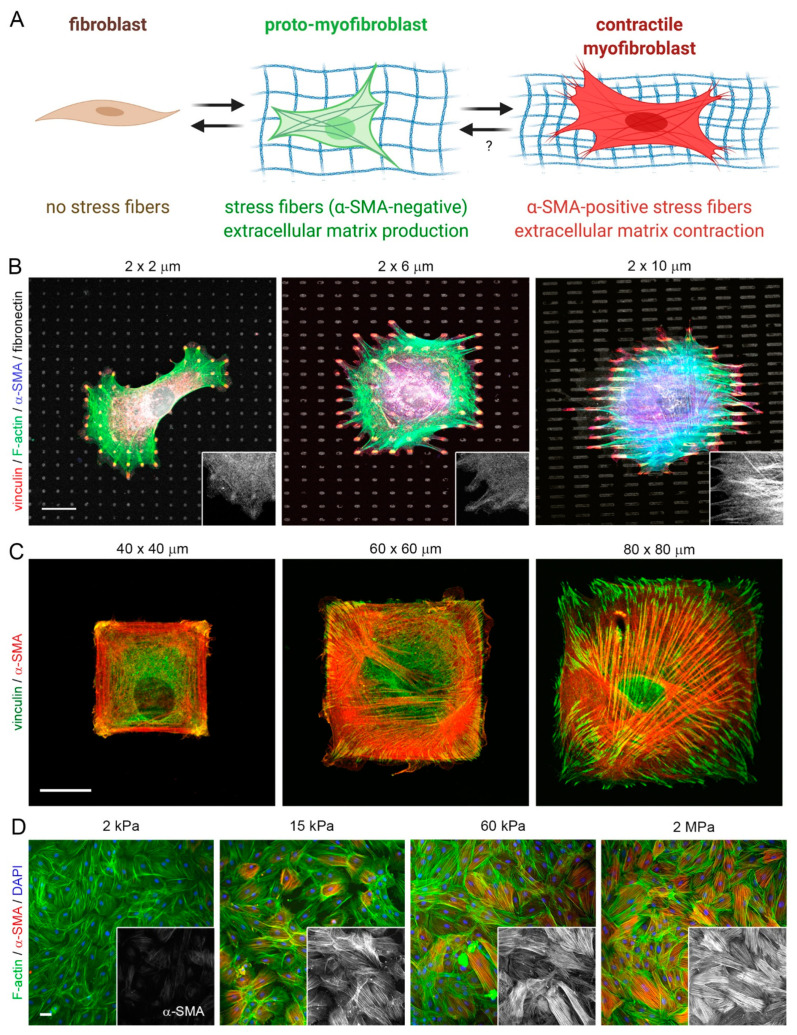
Control of fibroblast-to-myofibroblast activation by adhesion patterns and substrate stiffness. (**A**) Activation of non-contractile (‘quiescent’) tissue fibroblasts into highly contractile, α-SMA stress fiber forming myofibroblasts passes over consecutive activation stages, one of which is characterized by α-SMA-negative stress fibers. This so-called pro-myofibroblast produces extracellular matrix and is the prevalent fibroblast phenotype in conventional cell culture. Myofibroblast activation stages can be controlled in culture by altering substrate adhesion patterns or stiffness. (**B–D**) Myofibroblasts from various origins were seeded onto different 2D culture substrates, followed by immunostaining for various proteins, color-coded as indicated. Acute incorporation of the myofibroblast marker α-SMA into stress fibers is increasing as a function of (**B**) the size of small fibronectin attachment islets that accommodate single focal adhesions, (**C**) area of large fibronectin adhesive islands that house single cells, and (**D**) stiffness of fibronectin-coated silicone elastomer substrates (elastic modulus in kPa). All these factors directly affect the ability of myofibroblasts to develop intracellular stress (actomyosin contractility). Scale bars: 20 µm. Scheme produced with Biorender.com.

The formation of fibrotic capsules around implants was originally attributed to fibroblasts producing a highly fibrous and acellular ECM which contains collagen I/III, fibronectin, and proteoglycans [167]. However, it is now acknowledged that peri-implant tissue can additionally comprise multiple layers of contractile myofibroblasts that secrete and organize various ECM components—a hallmark of fibrosis [168,169]. ‘Myofibroblast’ denominates a pathophysiologically relevant fibroblast activation state that is characterized by neo-formation of stress fibers in tissues [56,57] (Figure 3). Stress fiber-mediated contraction confers to myofibroblasts the ability to repair tissues by organizing collagen ECM into mechanically resistant scar tissue. It is the excessive production, contraction, and crosslinking of collagen by transglutaminases [170,171,172], lysyl hydroxylases [173], lysyl oxidases [174], and lysyl oxidase-like enzymes [175] that result in fibrosis [176,177,178,179]. In vitro, all fibroblastic cells form stress fibers with stiff plastic or glass culture surfaces in the presence of serum and should thus be considered functional myofibroblasts [180]. Neo-expression of the smooth muscle actin (SMA) isoform α-SMA in stress fibers has become the most widely used molecular marker of myofibroblasts in culture and in vivo as it further enhances the contractile force of fibroblastic cells [181]. Recruitment of α-SMA into stress fibers is also an excellent indicator of acute intracellular and extracellular stress on different surfaces. During spreading on stiff culture surfaces, cytosolic α-SMA localizes to pre-formed β-cytoplasmic actin stress fibers only when spreading area is maximal, circular spreading symmetry is broken and myofibroblasts start to polarize into multipolar shapes by forming large focal adhesions [182]. Restriction of focal adhesion sizes by micropatterning stiff surfaces with adhesion sized fibronectin islets limits myofibroblast spreading, intracellular stress and α-SMA stress fiber incorporation [183]. The same acute effect is achieved by limiting cell spreading area using patterns of larger fibronectin islands [183,184] or culturing myofibroblasts on soft (<15 kPa) silicone substrates [181,183] (Figure 4).

All these principles are being applied to reduce myofibroblast activation at the surface of implant materials in culture systems and/or animal models of implant fibrosis [185]. In our own studies, we were able to suppress mechanical activation of myofibroblasts and implant fibrosis by covering MPa-stiff implant silicone surfaces with small focal adhesion micropatterns or soft (2 kPa) layers in rat and mouse models of subcutaneous implantation [69,186]. Others inhibited formation of large focal adhesions and fibroblast-to-myofibroblast activation using symmetrical arrays of hexagonal pits with micron-sized dimensions in hydrogels [105] or hierarchical micro/nano-topographical features in silicone materials [187]. A common theme of all these studies is that both, too little or much adhesion will be detrimental to implant integration and function and that both macrophage and fibroblasts have their sweet spots for implant acceptance. Surfaces that allow insufficient cell adhesion are perceived as body-foreign and elicit a strong inflammatory response. Surfaces allowing ‘unlimited’ cell adhesion and spreading signify large objects that activate hungry FBGCs and/or mechanically stimulate fibroblast transition onto fibrogenic myofibroblasts.

## 7. Outlook: Lessons to Learn from Anti-Fibrosis Strategies against Cell Mechanosensing?

Organ fibrosis comprises a group of heterogeneous connective tissue diseases that often lead to disruption of soft organ architecture and ultimately organ failure. Fibrosis is characterized by the excessive accumulation of disorganized and stiff ECM that often develops due to impaired wound healing as a result of the repetitive or chronic tissue injury [188,189,190]. Various factors, such as persistent infection, environmental exposure to toxin agents or smoke, genetic predisposition, and chronic inflammation contribute to development of tissue fibrosis [191]. Because fibroblast-driven encapsulation of implants by scar-like ECM shares features with organ fibrosis, anti-fibrotic therapies are being tested to preserve the function of implants that are threatened by capsular contractions [192], such as the lung fibrosis-approved drug pirfenidone [193] or using pan-αv integrin inhibitors [69,194]. Other studies targeted collagen type I synthesis using small interfering RNA which resulted in significant decrease the fibrous capsule thickness in a mouse implant model [195] (Table 1). We and others have already amply reviewed current strategies and therapies to counteract fibrosis in different organs [56,190,196,197,198]. For this review, we will limit our summary to strategies that aim to suppress myofibroblast actions but have not yet been considered or tested for implant fibrosis. The focus will be on mechanisms and molecules that are involved in the mechanical activation of myofibroblasts, which we identified as one major mechanism acting at the surface of stiff implants [69,186].

Aberrant ECM mechanosensing by fibroblastic cells and myofibroblast activation are associated with fibroproliferative disease conditions that involve changes in tissue environment and ECM composition [209,210,211]. Cells feel different mechanical stimuli through different cell surface receptors such as integrins, G-protein coupled receptors (GPCRs), or stretch-activated ion channels which activate mechanotransduction downstream signaling pathway [212]. One of the critical steps in the fibrotic phase of the FBR is fibroblast integrin engagement either with host proteins adsorbing to the implant, or to the provisional ECM developing around the object [213]. Integrin-based adhesion complexes mediate transmembrane signaling and intracellular responses to the environmental stimuli such as substrate stiffness [214,215,216,217,218] and thus represent prime anti-fibrosis targets [219,220,221]. In addition to the adhesion patterning approaches already discussed above, functionalization of implant materials with integrin-binding moieties, possibly including growth factor binding sites, has been proposed to guide a normal healing response and suppress the FBR and fibrosis [222,223].

Beyond their classical roles in direct mechanosensing, specific αv integrins are also involved in the activation of latent TGF-β1, a key factor in driving myofibroblast activation [196,224,225,226,227] (Figure 3). Fibroblasts and other cells secrete latent TGF-β1 in complex with its non-covalently bound latency-associated peptide (LAP), which in turn binds to the fibrillin protein family member latent TGF-β1-binding protein 1 (LTBP-1) in the ECM [228]. Integrins αvβ1, αvβ3, αvβ5, and αvβ6 have been demonstrated to physically interact with an RGD domain present in the LAP peptide and mechanically activate latent TGF-β1 by transmitting cytoskeletal forces to the LAP-TGF-β1 complex [229,230,231,232]. If the latent TGF-β1/LTBP-1 complex is integrated into a mechanically resisting matrix, cytoskeletal force exertion leads to a conformation change in the LAP-TGF-β1 ‘straitjacket’ configuration, resulting in activation and secretion of active TGF-β1 [226,227,233,234,235,236]. Our own recent study has shown that blocking αv integrins in a mouse model of implant fibrosis reduces TGF-β1 signaling at the implants surface and almost completely abolishes the formation of a fibrotic peri-implant tissue [69]. This study was inspired by seminal studies using similar approaches successfully in different experimental models of organ fibrosis [194,237].

In addition to driving pro-fibrotic cell programs thorough ‘canonical’ Smad signaling pathways, TGF-β1 also acts through the PI3K/Akt pathway, mitogen activated protein kinases (MAPKs) including Jun N-terminal kinases (JNKs), p38 MAPK (aka MAPK14), extracellular signal-regulated kinases (ERKs), and the Rho/Rho-associated protein kinase (ROCK) pathway [196,238,239]. Rho/ROCK signaling is a key regulatory pathway of actin-myosin contractility and central in controlling myofibroblast contraction [177,240,241], downstream of the engagement of the TGF-β1 receptor, and GPCR receptors such as proteinase-activated receptor 1 (PAR-1) or lysophosphatic acid receptor 1 (LPAR1) in a stressed environment [242,243,244,245]. Consequently, these receptor-signaling pathways are considered as anti-fibrosis targets [246]. Rho/ROCK signaling further regulates mechanical and receptor-mediated activation of Yes-associated protein 1 (YAP) and transcriptional coactivator with PDZ-binding motif (TAZ) [247,248]. YAP and TAZ are downstream co-activators of the Hippo signaling pathway, consisting of a cascade of mammalian sterile 20-like kinase 1/2 (MTS1/2) and large tumor suppressor kinase 1/2 (LATS1/2) [249,250]. YAP and TAZ are regulated by the activated and phosphorylated serine/threonine kinases LATS1/2, with YAP/TAZ phosphorylation resulting in cytoplasmic sequestration or proteasomal degradation [251]. Conversely, and relevant for fibrosis, MST1/2 activity is decreased by F-actin polymerization leading to inactivation of the Hippo complex. In this scenario and in direct dependence on force [252], non-phosphorylated YAP and TAZ translocate to the cell nucleus where they predominantly associate with transcription factors, such as TEA-domain family member (TEAD) but also Smad, p73, and Runt (RunX), to mediate transcription of fibrosis-related genes, including CCN2 and microRNA-21 [253,254,255,256]. YAP and TAZ are involved in myofibroblast activation and the onset of fibrosis in several organs, including lung, kidney, and liver [257,258,259,260,261]. Inhibition of selective GPCRs upstream of YAP/Taz signaling was shown to be effective in blocking myofibroblast activation and halt animal fibrosis [262].

Myocardin-related transcription factor (MRTF-A) is another mechanosensitive transcriptional effector that has been implicated in coordinating profibrotic signaling in myofibroblasts [209]. Upon mechanical stimulation and/or increased actin polymerization into stress fibers, MRTF-A becomes liberated from its inhibitory complex with G-actin in the cytoplasm, which then allows translocation of MRTF into the nucleus. In complex with serum response factor (SRF), nuclear MRTF-A regulate transcription of several cytoskeletal and ECM proteins that are associated with myofibroblast activation and fibrosis, including CCN2 and α-SMA [263,264,265,266]. Taken together, different mechanotransduction signaling pathways play distinct roles in myofibroblast activation and contraction in conditions of organ fibrosis. Targeting these pathways in the context of implanted solid materials that are almost always perceived as being stiff has great potential to alleviate peri-implant fibrosis.

## 8. Conclusions and Open Questions

Macrophage activation and fusion into FBGCs are hallmarks of the FBR and have been studied extensively because of their detrimental effect on medical device functions and surrounding tissue integrity. However, the consequences of chronic inflammation on fibroblastic cell populations in the vicinity of a—usually stiff—foreign body is less well documented. We and others have previously reviewed how macrophage-fibroblast interactions contribute to normal tissue repair and how dysregulation results in fibrosis; similar cross-talk is expected to take place in the environment of an implant [4,131]. Not unlike the sequalae taking place during organ fibrosis, chronic persistence of macrophages promotes activation of fibroblasts and expression of pro-fibrotic genes through paracrine signaling [267,268]. For instance, secretion and/or presentation of TGF-β1 by macrophages is a major contributor to the activation of fibroblasts and deposition of ECM in both, fibrosis and implant scarring [177,269] in a very localized niche environment [69,270]. In addition to secretory factors, the ECM itself plays a major role in mediating cellular behavior and intracellular communication. ECM guidance such as stiffness gradients and fibroblast induced contraction events in the ECM modulate macrophage-fibroblast communication [271,272,273,274]. However, not all fibrotic reactions to implanted materials appear to depend on macrophage actions. For example, macrophage depletion in a transgenic macrophage Fas-induced apoptosis (MaFIA) mouse model suppresses infiltration of inflammatory cells into hexamethylenediisocyanate-crosslinked dermal sheep collagen scaffolds which leads to an increased fibrotic capsule size around implants [275]. Furthermore, depletion of other immune cells including T-cells, NK cells, and mast cells depletion does not suppress the formation of the FBGC and fibrotic encapsulation of implants [276,277,278].

Stiff materials placed in provisional ECM create boundary conditions that are sensed over long distances by fibroblastic cells [279,280,281,282]. It is tempting to speculate that implants attract migrating fibroblasts, just by presenting an obstacle where lines of tension converge in provisional ECM. A review on the FBR from the perspective of a fibrosis researcher with a knack for cell biomechanics necessarily falls short in reporting on other exciting aspects of the FBR, such as the role of adaptive immune cells and design principles for of immunomodulatory biomaterials [130,135,283]. We also purposely excluded porous scaffold materials and degradation properties from our discussion which are intriguing both from a chemical composition and from a mechanical point of view—We hope we can be forgiven. Even in our core area of expertise, we did not even tempt to discuss a current hot topic in the fibroblast field: fibroblast origin and progeny [284]. The publication rate of single RNA sequencing studies classifying fibroblastic cells in various fibrotic organs and conditions has reached impressive if not overwhelming levels. However, we were not aware of a similar assessment of fibroblasts in peri-implant tissue—it will likely be different next week and then become topic of another review.

## Figures and Tables

**Table 1:** Current strategies to target the fibrotic phase of the FBR.

Inhibiting Compound	FBR Pathway Target	Implant Material	Implant Model	Species	Study Length (In Vivo)	References
Prolyl-4-hydroxylase inhibitors	Collagen synthesis	N/A	Intraocular implants	Human	19 months	[192,193,195,199,200]
Col1 siRNA (Nanofiber scaffold-mediated RNA interference)	Collagen synthesis	siRNA–poly(caprolactone-co-ethylethylene phosphate) nanofibers	Posterior dorsal areas	Rat	4 weeks	[195]
Rapamycin (mTOR) siRNA	Type I collagen synthesis	Poly(ethylene glycol) (PEG)-based hydrogel coatings	Subcutaneous siRNA-releasing device implantation	Mice	2 weeks	[199]
Halofuginone	Type I collagen synthesis	Silicone discs	Subcutaneous implantation	Rat	3 months	[201]
Relaxin, BMP-7, hepatocyte growth factor, SMAD7	TGF-β	Mock biosensors	Subcutaneous implantation	Rat	55 days	[192,193,202]
Pirfenidone	TGF-β and Collagen synthesis	Smooth and textured silicone implants	Submammary implantation	Rat	8 weeks	[193]
Masitinib	Tyrosine-kinase	Polyester fiber model	Subcutaneous implantation	Mice	4 weeks	[203]
Antisense oligonucleotides, cAMP, TNF	CCN2	polyether-polyurethane sponges	Subcutaneous implantation	Mice	14 days	[204,205]
Monoclonal antibodies specific for MMPs and TIMP-1	Pharmacological inhibition of MMP-1,-8,-13, and -18	In vitro human monocyte assay	In vitro	Human	In vitro	[206]
Pravastatin	Neovascularization and AMPK/mTOR pathway	Medical-grade Polyetheretherketone (SP)	Subcutaneous implantation	Mice	4 weeks	[207]
VEGF	Neovascularization	Commercial glucose sensors	Subcutaneous implantation	Mice	10 days	[208]

## Data Availability

Not applicable.
